# Abundant Expression of Guidance and Synaptogenic Molecules in the Injured Spinal Cord

**DOI:** 10.1371/journal.pone.0088449

**Published:** 2014-02-11

**Authors:** Anne Jacobi, Anja Schmalz, Florence M. Bareyre

**Affiliations:** 1 Institute of Clinical Neuroimmunology, Ludwig-Maximilians University Munich, Munich, Germany; 2 Munich Cluster for Systems Neurology (SyNergy), Munich, Germany; Heidelberg University Hospital, Germany

## Abstract

**Background:**

Spinal interneurons have emerged as crucial targets of supraspinal input during post-injury axonal remodelling. For example, lesioned corticospinal projections use propriospinal neurons as relay stations to form intraspinal detour circuits that circumvent the lesion site and contribute to functional recovery. While a number of the molecules that determine the formation of neuronal circuits in the developing nervous system have been identified, it is much less understood which of these cues are also expressed in the injured spinal cord and can thus guide growing collaterals and initiate synaptogenesis during circuit remodelling.

**Methodology/Principal Findings:**

To address this question we characterized the expression profile of a number of guidance and synaptogenic molecules in the cervical spinal cord of healthy and spinal cord-injured mice by *in situ* hybridization. To assign the expression of these molecules to distinct populations of interneurons we labeled short and long propriospinal neurons by retrograde tracing and glycinergic neurons using a transgenically expressed fluorescent protein. Interestingly, we found that most of the molecules studied including members of slit-, semaphorin-, synCAM-, neuroligin- and ephrin- families as well as their receptors are also present in the adult CNS. While many of these molecules were abundantly expressed in all interneurons examined, some molecules including slits, semaphorin 7a, synCAM4 and neuroligin 1 showed preferential expression in propriospinal interneurons. Overall the expression pattern of guidance and synaptogenic molecules in the cervical spinal cord appeared to be stable over time and was not substantially altered following a midthoracic spinal cord injury.

**Conclusions:**

Taken together, our study indicates that many of the guidance and synaptogenic cues that regulate neuronal circuit formation in development are also present in the adult CNS and therefore likely contribute to the remodelling of axonal connections in the injured spinal cord.

## Introduction

For successful wiring of the nervous system axons need to navigate and establish synaptic contacts with their proper target cells. Work in the developing nervous system has established that this process is regulated by target derived guidance and synaptogenic cues (for review see [1], [2]). A number of the molecules that can guide growing axons in the developing nervous system have been identified and include among others netrins [3], semaphorins [4], slits [5] and ephrins [6]. Similarly, molecules that can facilitate pre- and postsynaptic differentiation following axon-target contact have been studied in neuronal development. Among these, synCAMs [7] and neuroligins [8], for example, can act as pre-synaptic organizers while neurexins [9], and ephrinBs [10] are postsynaptic organizers. To what extend these molecules also regulate pathfinding and synapse formation of re-growing axons in the damaged adult nervous system is so far only incompletely understood.

During the recent years, it has become increasingly clear that new axonal connections are not only formed during development but also following nervous system injury. For example, we and others have shown that the corticospinal tract (CST) undergoes extensive remodelling following spinal cord injury [11]–[14]. A key element of this remodelling process is the formation of intraspinal detour circuits [11], [13]. For detour circuits to form, the hindlimb CST sprouts new collaterals in response to a midthoracic dorsal hemisection. These collaterals then enter the gray matter of the cervical spinal cord and contact different populations of spinal interneurons including C3–C4 short propriospinal neurons (SPSN) - which are important for visually-guided target reaching with the forelimb [15] - and C3–C5 long propriospinal neurons (LPSN) - which contribute to locomotion and in particular mediate the coupling of forelimbs and hindlimbs during walking [16]. Initially CST collaterals equally contact long and short propriospinal neurons, however over time contacts onto SPSN are partially removed while contacts onto LPSN are refined and maintained [11], [13]. LPSN in turn increase their projections onto lumbar motoneurons and thereby complete an intraspinal detour circuit that can relay information from hindlimb motor cortex to the lumbar spinal cord. The importance of intraspinal detour circuits has been further emphasized by a number of subsequent studies that demonstrate that similar detour circuits (i) mediate the recovery of the supraspinal control of stepping after spinal cord injury [12], (ii) also form in response to inflammatory insults to the spinal cord [17], [18], and (iii) are the target of therapeutic strategies that can promote remarkable recovery of locomotor function in rodents [19], [20]. While the importance of intraspinal detour circuits for functional recovery is thus well-established, it is unclear how the initiation and stabilization of the synaptic contacts that form the new circuits is regulated. To identify candidate cues that can guide the formation of intraspinal detour circuits we investigated the expression pattern of a number of membrane-bound guidance and synaptogenic molecules in the cervical spinal cord of healthy mice and spinal-cord injured mice by *in situ* hybridization. In particular we assessed the expression in the following populations of spinal interneurons: (i) C3–C4 SPSN, (ii) C3–C5 LPSN and (iii) glycinergic neurons which are located in similar spinal laminae as propriospinal neurons and served as control population. Our results show that members of the slit-, semaphorin-, synCAM-, neuroligin- and ephrinB- families are abundantly expressed in spinal interneurons both before and after spinal cord injury. While most of these molecules are equally expressed in the different interneuronal populations, some molecules like slits, semaphorin 7a and neuroligin 1 are present in many propriospinal neurons but in only few glycinergic interneurons. These results suggest that similar molecular mechanisms might regulate the initial formation of circuits in development and their re-formation after injury.

## Materials and Methods

### Ethics Statement

All animal experiments conformed to the institutional guidelines and were approved by the Animal Study Committee of the Regierung von Oberbayern. Approval ID: 55.2-1-54-2531-127-05.

### Mice

Adult mice between 6 and 12wks of age were used in this study. C57/Bl6 mice (Janvier SAS) were used to study stereotactically labeled long and short propriospinal neurons. *GlyT2*- EGFP mice that express enhanced green fluorescent protein (EGFP; [21]) under the control of the GlyT2 promoter were used to label inhibitory glycinergic neurons.

### Surgery procedure

For hemisection procedures mice were anesthetized by i.p. injections of ketamine/xylazine (ketamine 87 mg/kg, xylazine 13 mg/kg). The dorsal spinal cord was exposed by a laminectomy at thoracic level 8 (Th8) and a dorsal hemisection which completely interrupts the main dorsal and minor dorsolateral CST was made with fine iridectomy scissors as previously described [13]. After surgery the mice were kept on a heating pad (38°C) until fully awake and treated with Metacam (0.05 mg/kg, Boehringer Ingelheim) for two more days. Spinal cord and cortex that were used for further analysis were derived from the same mice.

### Retrograde labelling of propriospinal neurons and cortical projection neurons

To co-localize the *in situ* hybridization (ISH) signal with propriospinal neurons, these neurons were retrogradely-labeled two weeks before sacrifice for all time points investigated. One µl of Fluoro-Emerald (10% in 1 x PBS, Life Technologies) was stereotactically injected with a glass capillary filled into the lower thoracic cord (Th12) to label LPSN and into the lower cervical cord (C8-Th1) to label SPSN on both sides of the spinal cord (±1.0 mm lateral from spinal midline, depth 1.0 mm). The micropipette remained in place for 2min after completing the injection. To co-localize the ISH signal with the cortical projection neurons of the transected CST, these neurons were retrogradely-labeled 7 days before sacrifice. Briefly, after laminectomy at thoracic level 8 of the spinal cord, 0,5 µl of TexasRed® (5% in 0.1 M PB, Life Technologies) was stereotactically injected rostrally to the lesion with a glass capillary into each side of the spinal cord (±0,2 mm lateral from spinal midline, depth 0.3 mm). The micropipette remained in place for two minutes after completing the injection to avoid backflow. After retrograde labelling mice were kept on a heating pad (38°C) until fully awake and treated with Metacam for 2 more days.

### Tissue preparation

Animals were deeply anesthetized with isoflurane and perfused transcardialy with saline solution followed by 4% paraformaldehyde (PFA) in 0.1 M phosphate buffer (PBS). After post-fixation in 4% PFA at 4°C overnight the spinal cord and brains were dissected, incubated in 30% sucrose for 2–3 d, frozen and stored at –20° until use.

**Immunohistochemistry (IHC).** To assess the presence of the ISH signal in NeuN positive neurons, the cervical spinal cord was sectioned in coronal orientation (30 µm) with a cryostat (Leica CM1850) and sections were then washed three times for 10min in 1x PBS. All solutions used for the IHC contained DEPC to prevent degradation of target RNAs for later ISH. After washing the sections were blocked for 45min in 1x PBS containing 10% horse serum and 0.1% Triton. The primary antibody anti-NeuN (1∶500; Millipore MAB377) was incubated in 1x PBS solution containing 0.1% Triton and 2.5% goat serum overnight at 4°. On day 2 the tissue was washed three times for 10min in 1x PBS before the application of the secondary antibody (1:500, AlexaFluor 488 goat anti-mouse; Life Technologies). After 3hrs of incubation the tissue was washed three times for 10min in 1x PBS and mounted in VectaShield.

### In situ Hybridization (ISH)

Spinal cord tissue (cervical region C2–C5) and brain tissue (Bregma –1.06 till –1.70) were sectioned in coronal orientation (50 µm thick) with a cryostat (Leica CM1850) and washed two times for 10min in 2X SSC (from 20X stock solution containing 3M NaCl and 0,3M Na Citrate). All steps were carried out with DEPC treated solutions to prevent degradation of target RNAs. Before the prehybridization step, the sections were incubated in a 1∶1 mixture of 2X SSC and hybridization buffer (50% Formamide, 5X SSC, 5X Denhardt’s solution (Sigma-Aldrich D2532), 250 µg/ml yeast tRNA, 500 µg/ml salmon sperm DNA) for 15min at RT. Afterwards the sections were incubated for 1hr in hybridization buffer at the appropriate (pre-) hybridization temperatures for each probe (see Table 1). For hybridization, the probe (200–400 ng/ml in hybridization buffer) was heated for 10min at 80°C, applied to the tissue and incubated overnight in an oven (for temperatures see Table 1). Sections were then rinsed at RT in 2X SSC and washed in decreasing concentration of SSC (2X to 0.1X SSC at hybridization temperature) before applying an alkaline-phosphatase-conjugated sheep anti-digoxigenin antibody, Fab fragments (1:2000; Roche Diagnostics) in blocking buffer overnight at 4°C. Alkaline phosphatase activity was detected using nitroblue tetrazolium chloride (337.5 mg/ml) and 5-Bromo-4-chloro-3-indolyl phosphate (175 mg/ml) (Carl Roth). The sections were washed in ddH_2_O after the staining procedure. When applied, the fluorescent Nissl stain Neurotrace 435 was applied for 2h at RT, the sections were washed and mounted with Gel Mount (Sigma Aldrich).

**Table 1:** Hybridization and pre-hybridization temperatures for the different probes used in the study.

	Origin of the probe	Prehybridization Temperature	Hybridization Temperature	Washing Temperature
Slit-1	rat	45°C	48°C	55°C
Slit-2	rat	45°C	48°C	55°C
Slit-3	rat	45°C	48°C	55°C
Robo-1	rat	50°C	54°C	60°C
Robo-2	rat	50°C	54°C	60°C
Robo-3	rat	50°C	58°C	65°C
Sema6A	mouse	50°C	50°C	60°C
Sema7A	rat	50°C	55°C	65°C
PlexinA2	mouse	48°C	48°C	55°C
PlexinC1	mouse	50°C	55°C	65°C
SynCAM1	mouse	55°C	60°C	65°C
SynCAM3	mouse	55°C	65°C	65°C
SynCAM4	mouse	55°C	60°C	65°C
NL1	mouse	55°C	55°C	55°C
NL4	mouse	55°C	55°C	55°C
EphB2	mouse	50°C	52°C	55°C
EphrinB1	mouse	50°C	55°C	55°C

### Imaging and image processing

As the ISH procedure interferes with the fluorescent labels we analyzed fluorescence and ISH signals using a two-step approach. For visualizing the co-localization of ISH signals and NeuN immunolabelling, we first imaged sections immunostained for NeuN using a confocal microscope (FV1000, Olympus). The sections were then unmounted, ISH was performed as described above before the same sections were then re-imaged with a confocal microscope using bright field illumination. Both images (that were acquired with the same magnification) were overlaid in Photoshop (Adobe) and a number of anatomical landmarks including the central canal and the ventral and dorsal border between the gray and white matter were used as fiduciary marks to co-register the images and adjust for tissue shrinkage due to the ISH process. For imaging retrogradely-labeled CST neurons, we first imaged the fluorescence signals using a confocal microscope (FV1000, Olympus) using standard filter settings before we unmounted the sections, performed ISH and image alignment as described above. To assess the presence of ISH signals in transgenically-labeled glycinergic interneurons, we first imaged the fluorescence signals using an epifluorescence Olympus IX71 microscope using standard filter settings before we unmounted the sections, performed ISH and image alignment as described above. Retrogradely-labeled propriospinal neurons were imaged using an epifluorescence Olympus IX71 concomitantly for fluoro-emerald and ISH signals as the ISH did not interfere with this fluorescent label.

### Image analysis and cell counts

Retrogradely-labeled cortical projection neurons and propriospinal neurons were assessed under the fluorescent microscope by alternating between the fluorescence and the bright field. Glycinergic neurons were assessed on the acquired images.

To determine the proportion of cortical projection neurons that express a given molecule, we counted all retrogradely-labeled neurons on every third sections of the cortex (n = 3 mice). Sections were assessed from anterior to posterior and the analysis began with the first section in which retrogradely-labeled CST neurons appeared. To determine the proportion of glycinergic neurons that express a given molecule, three sections at C3/C4 and three sections at C5 were randomly selected. Then, all glycinergic neurons in lamina VI to IX (which are the laminae in which long and short propriospinal neurons are located) were assessed (n = 3 mice). To determine the proportion of long and short propriospinal neurons that express a given molecule, all retrogradely-labeled neurons located from C3 to C5 were counted until the number of cells reached 30 per animal (about 10 sections per animal, n = 3 mice) taking the first section as the section in which propriospinal neurons were first detected. Results were expressed as a ratio of the number of double-labeled neurons compared to the total number of assessed neurons. All counts were performed by an independent blinded-observer. To assess co-localization we used the following evaluation criteria: A cell was considered countable when the contour of its soma could be clearly identified either based in the retrograde label or based on the fluorescent transgenic label. ISH signals were considered to be overlaying when they followed the contour of the soma and did not extend beyond it.

For generating the rating in Table 2 and 3, we first defined the area for the analysis e.g. laminae VI to IX for the spinal cord or in the cortex at –1.3 from bregma starting at +/– 1 mm from the midline. To assess the expression in different cortical layers we used a box of 35 µm^2^ that was overlaid on layer 1, or 2 or 3 or 4 or 5 or 6 of the cortex. We then set the threshold for detection and measured the grayscale intensity of the selected area with the ImageJ Measurement Tool. Values below 500 were defined as not detected; Values between 500 and 1500 were defined as +; Values between 1500 and 2500 were defined as ++; Values between 2500 and 3500 were defined as +++; Values above 3500 were defined as ++++.

**Table 2:** Distribution and intensity of the ligands in the unlesioned cervical spinal cord of adult mice.

Area	Slit-1	Slit-2	Slit-3	Sema6a	Sema7a	SynCam1	SynCam3	SynCam4	NL1	NL4	EphB2	EphrinB1
Gray Matter												
Laminae I – IV	++	+++	+	+	++	++	+	++	+	++	+++	+
Laminae V	++	++++	++	+++	+++	++++	+++	+++	++	++	+++	++
Laminae VI – IX	+++	++++	++	++++	+++	+++	++++	+++	+++	++	+++	++
White Matter												
Dorsal Column	+	+	– .	–	+	–	–	+	–	–	+	+
Ventral Funiculus	+	+	–	–	+	–	–	+	–	–	–	+

Relative intensities were estimated by visual comparison with sense probe in situ hybridization slides: +: weak; ++: moderate; +++: strong; ++++: very strong; –: not detected.

**Table 3:** Distribution and intensity of the receptors in the unlesioned adult mouse cortex.

Area	Robo1	Robo2	Robo3	PlexinA2	PlexinC1	SynCam1	SynCam3	SynCam4	EphB2	EphrinB1
LayerI	+	–	–	–	–	+	–	–	–	–
Layer II	+	+	+	+	+	++	++	++	++	++
Layer III	++	++	–	++	++	++	++	+	++	++
Layer IV	++	++	–	–	–	++	+	+++	–	–
Layer V	++++	+++	++	++	+++	++	++	++++	+++	+++
Layer VI	++	+	+	+	–	++	+	++	–	–

Relative intensities were estimated by visual comparison with sense probe in situ hybridization slides: +: weak; ++: moderate; +++: strong; ++++: very strong; –: not detected.

### Statistical analysis

Results are given as mean ± SEM unless indicated otherwise. Data were analyzed in GraphPad Prism5.01 software using a two-way ANOVA (factors: time and interneuron-type) followed by Bonferroni post hoc tests. Significance levels was taken with p<0.01.

## Results

### Expression of the repulsive axon guidance cues slit-1, -2 and -3 and their receptors in the adult CNS

**Figure 1:**
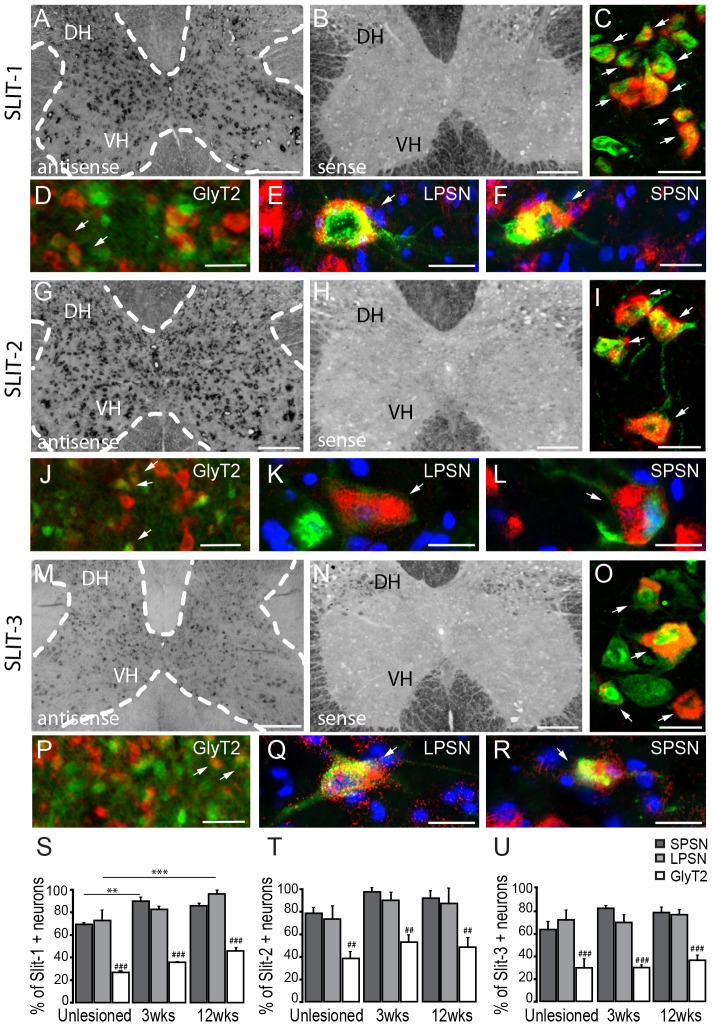
*In Situ* hybridization pattern of Slit-1,-2,-3 in the cervical spinal cord. *In situ* hybridization of slit-1 (A-F), slit-2 (G-L), slit-3 (M-R) mRNA in the unlesioned cervical spinal cord. Strong signals for slit-1 (A) and slit-2 (G) are detected with the anti-sense probe in cervical interneurons and motoneurons while slit-3 (M) shows a weaker signal. No signals are detected with the sense probes for slit-1 (B), slit-2 (H) or slit-3 (N). (C, I, O) Epifluorescence images of double-labeled neurons in the ventral horn (NeuN: green; *In situ* signal: red). (D-F) Co-localization of slit-1 mRNA in glycinergic neurons (D; GlyT2: green; slit-1: red), LPSN (E; LPSN: green; slit-1: red; NT435: blue) and SPSN (F; SPSN: green; slit-1: red; NT435: blue) in the cervical spinal cord. (J-L) Co-localization of slit-2 mRNA in glycinergic neurons (J; GlyT2: green; slit-2: red), LPSN (K; LPSN: green; slit-2: red; NT435: blue) and SPSN (L; SPSN: green; slit-2: red; NT435: blue) in the cervical spinal cord. (P-R) Co-localization of slit-3 mRNA in glycinergic neurons (P; GlyT2: green; slit-3: red), LPSN (Q; LPSN: green; slit-3: red; NT435: blue) and SPSN (R; SPSN: green; slit-3: red; NT435: blue) in the cervical spinal cord. (S-U) Quantification of the proportion of glycinergic neurons, LPSN and SPSN expressing slit-1 (S), slit-2 (T) and slit-3 (U) in the unlesioned and lesioned cervical spinal cord. Scale bars in A,B,G,H,M,N, 250 µm; Scale bars in C,I,O, 25 µm; Scale bars in D-F,J-K,P-R, 25 µm.

The process of axon pathfinding is mediated by a number of guidance molecules, among them slits (slit-1,-2,-3) and their receptors (robo-1,-2,-3), which have been shown to have a repulsive effect on axons during development [22]–[23] and have been proposed to restrict axonal growth at the lesion site following spinal cord injury [24]. To assess whether slits can also regulate axon growth during axonal remodelling distant from the lesion site we first investigated the expression of slit family members in the unlesioned mouse spinal cord by *in situ* hybridization. Hybridization with the anti-sense probe shows that slit-1, slit-2 and slit-3 mRNAs are detected in all laminae of the cervical gray matter (Fig. 1A,G,M) while hybridization with the sense probe showed no signal (Fig. 1B,H,N). In particular, slit-1 and slit-2 show high expression levels throughout the spinal gray matter and in particular in the ventral horn (Table 2) while slit-3 mRNA seems to be expressed at a lower level. Analysis after counterstaining with NeuN suggests that slits are expressed by neurons including by interneurons and motoneurons (Fig. 1C,I,O). To better understand which populations of spinal interneurons express slit mRNA, we visualized glycinergic interneurons using a transgenic label (Fig. 1D,J,P) and LPSN (Fig. 1E,K,Q) and SPSN (Fig. 1F,L,R) using retrograde labelling . Our results show that, 60 to 80% of all propriospinal neurons but only about 30% of glycinergic interneurons express slit-1, -2 and -3 (Fig. 1S-U). While overall this expression pattern is rather unchanged in the cervical spinal cord of mice at 3 and 12 weeks after injury, there is a moderate but significant increase in the proportion of propriospinal neurons that expressed slit-1 or slit-3 after injury (Fig. 1 S-U).

**Figure 2:**
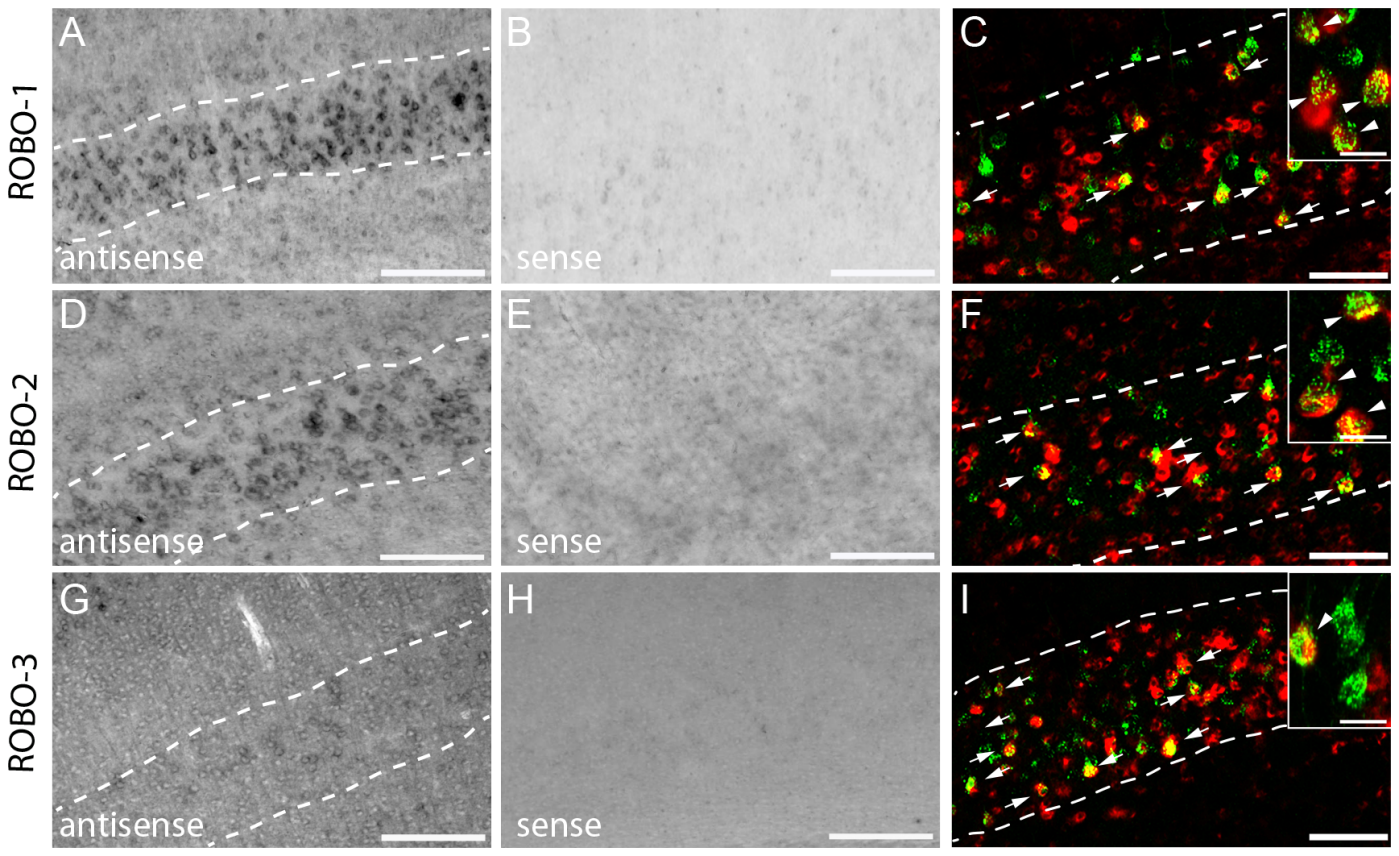
*In Situ* hybridization pattern of Robo-1,-2,-3 in the cortex. *In situ* hybridization of Robo-1 (A), Robo-2 (D) and Robo-3 (G) mRNA in the cortex. No signals are detected with the sense probes (B: Robo-1; E: Robo-2; H: Robo-3). Dotted lines in A, D, and G represent layer V. (C,F, I) Epifluorescence images of double-labeled neurons of layer V (Retrogradely-labeled CST neurons: green; *In situ* signal: red).Scale bars in A,B,D,E,G,H: 100 µm; Scale bars in C,F,I: 50 µm (25 µm in insets).

To determine whether the corticospinal collaterals that enter the spinal gray matter can respond to slits expressed by spinal interneurons we examined the expression of the corresponding slit-receptors (robo-1, -2, -3) in the mouse cortex by *in situ* hybridization . In the cortex, robo-1 can be detected in layer I to VI, with its strongest expression in the cells of layer V (Table 3 and Fig. 2A). Robo-2 is expressed from layer II to VI in the cortex, with a slightly more intense labelling in layer V (Fig. 2D). Additionally, robo-3 mRNA is detectable in layer II, V and VI although the expression level is very low (Fig. 2G). Specificity of the staining was validated by hybridization of the tissue with the sense probe which showed no signals (Fig. 2 B,E,H). Retrograde labelling with Texas Red® revealed that 90,2±3,4% of CST projection neurons in layer V express robo-1, 55,2±5,2% of CST projection neurons express robo-2 and that 26,4±1,9% of CST projection neurons express robo-3 (Fig. 2 C,F,I).

### Expression of the guidance cues semaphorin 6a (Sema6a) and semaphorin 7a (Sema7a) and their receptors plexin A2 and plexin C1 in the adult CNS

**Figure 3:**
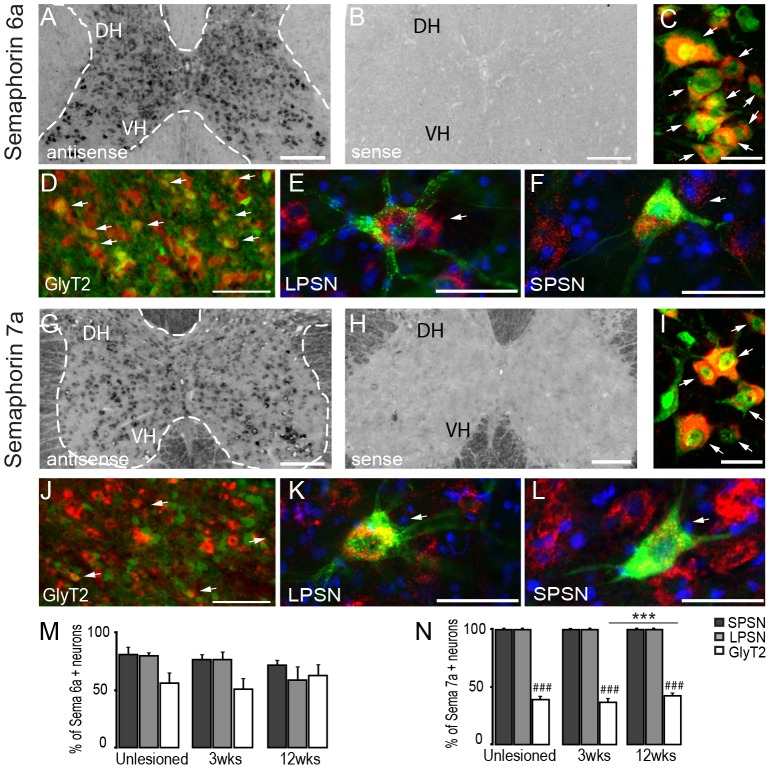
*In Situ* hybridization pattern of Semaphorin 6a and Semaphorin 7a in the cervical spinal cord. *In situ* hybridization of Semaphorin 6a (A) mRNA in the unlesioned cervical spinal cord. Strong signal for Semaphorin 6a (A) is detected with the anti-sense probe. No signal is detected with the sense probe (B). (C) Confocal picture of double-labeled neurons in the ventral horn (NeuN: green; Semaphorin 6a: red). (D-F) Co-localization of Semaphorin 6a mRNA in glycinergic neurons (D; GlyT2: green, Semaphorin6a: red), LPSN (E; LPSN: green, Semaphorin 6a: red, NT435: blue), and SPSN (F; SPSN: green, Semaphorin 6a: red, NT435: blue) in the cervical spinal cord. *In situ* hybridization of Semaphorin 7a (G) mRNA in the unlesioned cervical spinal cord. Moderate signals for semaphorin 7a in inter- and motoneurons for Semaphorin 7a (G) is detected with the anti-sense probe. No signal is detected with the sense probes (H). (I) Confocal image of double-labeled neurons in the ventral horn (NeuN : green; Semaphorin 7a: red). (J-L) Co-localization of Semaphorin 7a mRNA in glycinergic neurons (D; GlyT2: green, Semaphorin 7a: red), LPSN (E; LPSN: green, Semaphorin 7a: red, NT435: blue), and SPSN (F, SPSN: green, Semaphorin 7a: red, NT435: blue) in the cervical spinal cord. (M, N) Quantification of the number of GlyT2, LPSN and SPSN expressing Semaphorin 6a (M) and semaphorin 7a (N) in unlesioned and lesioned cervical spinal cord. DH: dorsal horn; VH: ventral horn. Arrows in D and J show double-labeled glycinergic neurons. Scale bars in A, B, G, H: 250 µm; Scale bars in C, I: 25 µm; Scale bars in E, F, K, L: 25 µm; Scale bars in D, J: 50 µm.

The repulsive membrane associated Sema6a has been shown to control axon guidance in different parts of the nervous system [25]-[27] and specifically to affect the growth of the developing CST at multiple choice points [28]. We now investigated the expression pattern of Sema6a in the cervical spinal cord of healthy mice by *in situ* hybridization. While Sema6a mRNA is specifically present throughout the spinal gray matter (Fig. 3A,B) the hybridization signals are more intense in the middle and ventral laminae (IV – IX) of the spinal cord than in the dorsal horn (Fig. 3A and Table 2). Morphology analysis after counterstaining with NeuN suggests that in the gray matter Sema6a is primarily expressed by neurons and in particular by interneurons (Fig. 3C). Sema6a mRNA is also present in cells in the dorsal and ventral white matter (Table 2), consistent with the reported expression of Sema6a in oligodendrocytes, [25].

Analysis of different interneuronal populations revealed that the majority of glycinergic interneurons as well as LPSN and SPSN contain Sema6a mRNA (Fig. 3D-F) both before and at 3 and 12 weeks after a thoracic spinal cord injury (Fig. 3M).

In contrast to Sema6a, semaphorin7a (Sema7a) is an attractive guidance cue that supports axonal growth [29]. In order to determine the expression pattern of Sema7a, we hybridized a Sema7a anti-sense probe to sections from the cervical spinal cord of healthy mice. We can show that Sema7a is specifically expressed in all laminae of the spinal cord (I – IX; Table 2 and Fig. 3G,H) with the strongest expression in ventral and intermediate laminae (Table 2). Cells that expressed Sema7a morphologically resembled interneurons (Fig. 3I) and further anaylsis revealed that all long and short propriospinal neurons but only about 40% of glycinergic interneurons expressed Sema7a both in the healthy spinal cord as well as 3 and 12 weeks after spinal cord injury (Fig. 3J-L, N).

**Figure 4:**
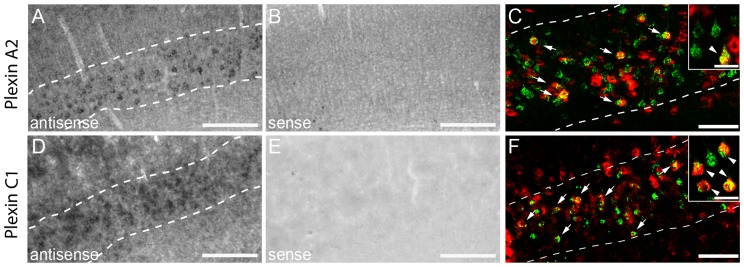
*In Situ* hybridization pattern of plexin A2 and plexin C1 in the cortex. *In situ* hybridization of PlexinA2 in the cortex (A). No signal is detected with the Sema6a sense probe (B). Dotted lines in A represent layer V of the cortex. (C) Confocal image of double-labeled neurons of layer V (retrogradely-labeled CST projection neurons, green; plexin A2, red). *In situ* hybridization of PlexinC1 in the cortex (D). No signal is detected with the Sema7a sense probes (E). Dotted lines in D represent layer V of the cortex. (F) Confocal picture of double-labeled CST projection neurons identified by retrograde tracing (retrogradely-labeled CST projection neurons, green; plexin C1, red). Scale bars in A,B,D,E: 100 µm; Scale bars in C, F: 50 µm (25 µm in insets).

To determine whether corticospinal axons can integrate attractive or repulsive signals from Sema6a or 7a, we detected the mRNA coding for the main receptor of Sema6a, plexinA2, and the receptor for Sema7a, plexinC1 in the mouse cortex by *in situ* hybridization. Our results show specific expression of plexinA2 in layers II-III and V-VI of the cortex (Fig. 4.A,B and Table 3) and specific expression of plexinC1 in in layers II-III and V (Fig. 4D,E and Table 3) Notably, both plexinA2 and plexinC1 are expressed in retrogradely-labeled CST projection neurons in layer V(plexinA2: 54,0±3,65%; plexinC1: 70,0±2,00%) (Fig. 4C,F).

### Expression of the bidirectional synaptogenic cues SynCAM1, SynCAM3 and SynCAM4 in the adult CNS

**Figure 5:**
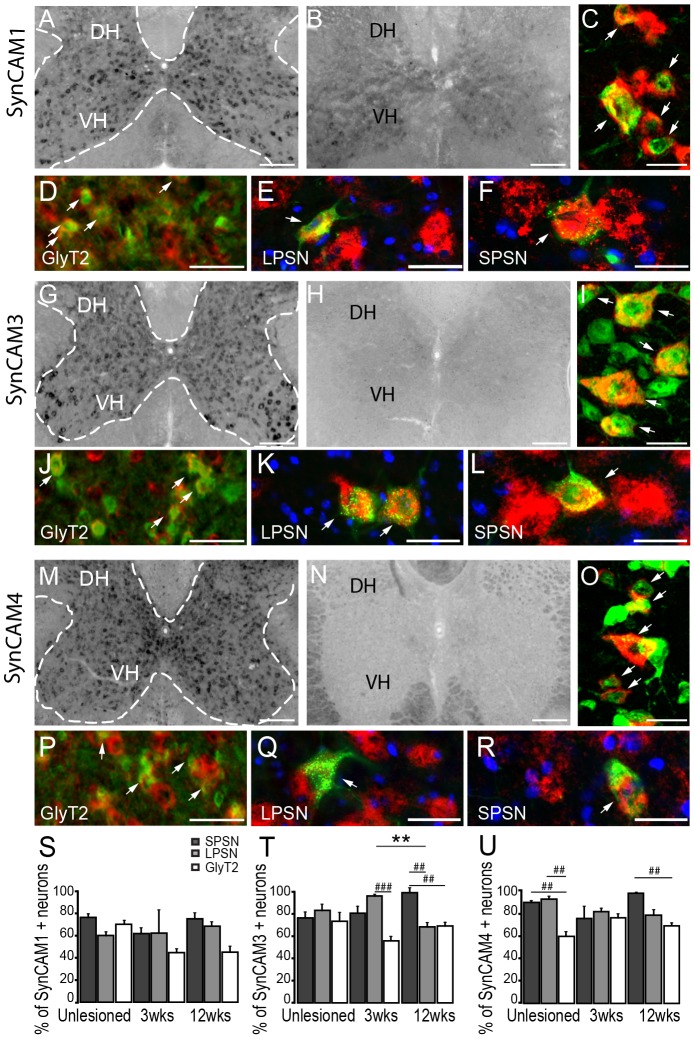
*In Situ* hybridization pattern of SynCAM1, SynCAM 3, SynCAM 4 in the cervical spinal cord. *In situ* hybridization of SynCAM1 (A) mRNA in the unlesioned spinal cord. Strong signal for SynCAM1 (A) is detected with the anti-sense probe. No signal is detected with the sense probe (B). (C) Epifluorescence picture of double-labeled neurons in the ventral horn (NeuN: green; SynCAM1: red). (D-F) Co-localization of SynCAM1mRNA in glycinergic neurons (D; GlyT2: green; SynCAM1: red), LPSN (E; LPSN: green; SynCAM1: red; NT435: blue) and SPSN (F; SPSN: green; SynCAM1: red; NT435: blue) in the cervical spinal cord. *In situ* hybridization of SynCAM3 (G) in the unlesioned spinal cord. Moderate signal for SynCAM3 (G) mRNA is detected with the anti-sense probe. No signal is detected with the sense probe (H). (I) Epifluorescence picture of double-labeled neurons in the ventral horn (NeuN: green; SynCAM3: red). (J-L) Co-localization of SynCAM3 in glycinergic neurons (J; GlyT2: green; SynCAM3: red), in LPSN (K; LPSN: green; SynCAM3: red; NT435: blue) and SPSN (L, SPSN, green; SynCAM3, red; NT435: blue) in the cervical spinal cord. *In situ* hybridization of SynCAM4 (M) in the unlesioned spinal cord. Strong signal for SynCAM4 (M) is detected with the anti-sense probe. No signal is detected with the sense probe (N). (O) Epifluorescence image of double-labeled neurons in the ventral horn (NeuN: green; SynCAM4: red). (P-R) Co-localization of SynCAM4 in glycinergic neurons (P; GlyT2: green; SynCAM4: red), in LPSN (Q; LPSN, green; SynCAM4, red; NT435: blue) and SPSN (R; SPSN, green; SynCAM4, red; NT435: blue) in the cervical spinal cord. (S-U) Quantification of the number of GlyT2 neurons, LPSN and SPSN expressing SynCAM1 (S), SynCAM3 (T) and SynCAM4 (U) in unlesioned and lesioned cervical spinal cord. DH: dorsal horn; VH: ventral horn. Arrows in D, J and P show double-labeled glycinergic neurons. Scale bar in A,B,G,H,M,N: 250 µm; Scale bar in C,I,O: 25 µm; Scale bars in D,J,P: 40 µm; Scale bars in E,F,K,L,Q,R: 25 µm.

Once the newly growing collaterals have been guided to their target cells, they need to make appropriate synaptic connections. During development this process is regulated by molecules like the synaptic cell adhesion molecules (SynCAM) that promote synapse formation and maturation [7]. In order to determine the expression pattern of these bidirectional synaptic cues we analysed the mRNA expression of SynCAM1, 3 and 4 in the cervical spinal cord. In adult healthy mice SynCAM1, 3, 4 expression appears to be limited to the gray matter with particular strong signals seen in the ventral horn (Fig. 5A,B,G,H,M, N and Table 2). Morphology analysis after counterstaining with NeuN indicates that SynCAMs are primarily expressed by neurons including by interneurons and motoneurons (Fig. 5C,I,O). Expression in motoneurons was confirmed by ChAT immunostaining (data not shown).

In line with these findings, mRNAs for SynCAM1, 3 and 4 can be detected in the majority of LPSN, SPSN and glycinergic interneurons (Fig. 5D-F,J-L,P-R,S-U). Mostly similar expression patterns were observed in the cervical spinal cord of healthy mice and the cervical cord of mice perfused at 3 or 12 weeks following a thoracic spinal cord injury (Fig. 5 S-U).

**Figure 6:**
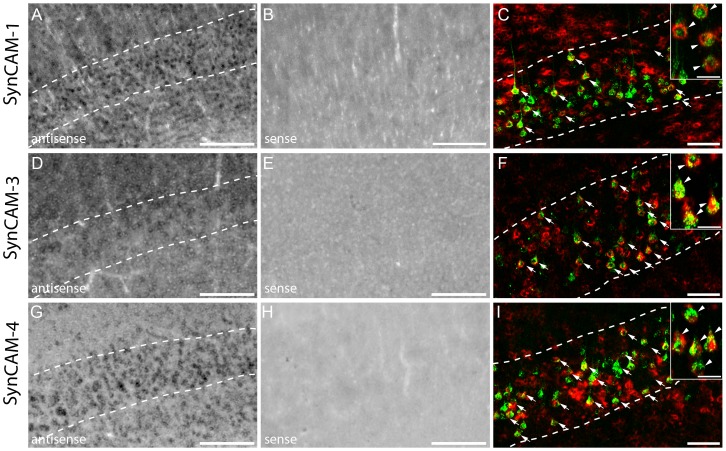
*In Situ* hybridization profile of SynCAM1, SynCAM 3, SynCAM 4 in the cortex. Profile of expression of SynCAM1 (A) mRNA in the cortex. No signal is detected with the SynCAM1 sense probe (B). Dotted lines in A represent layer V of the cortex. (C) Confocal image of double-labeled neurons of layer V (retrogradely-labeled CST neurons: green; SynCAM1: red). Profile of expression of SynCAM3 (D) mRNA in the cortex. No signal is detected with the sense SynCAM3 probe (E). Dotted lines in D represent layer V of the cortex. (F) Confocal image of CST projection neurons (retrogradely-labeled CST neurons: green, SynCAM3: red). ). Profile of expression of SynCAM4 (G) mRNA in the cortex. No signal is detected with the sense SynCAM4 probe (H). Dotted lines in G represent layer V of the cortex. (I) Confocal image of double-labeled CST projection neurons (retrogradely-labeled CST projection neurons: green; SynCAM4: red). Scale bar in A, B, D, E, G, H: 100 µm; Scale bar in C,F,I: 50 µm (25 µm in insets).

Further analysis showed that SynCAM1 and SynCAM4 and to a lesser extent SynCAM3 are also expressed in the cortex (Table 3 and Fig. 6A,B,D,E,G,H). In all cases expression seems to be strongest in layer V neurons. In particular, we show that 69,8±6,5% of retrogradely-labeled layer V CST projection neurons express SynCAM1; 73,1±7,3% SynCAM3 and 94,7±0,3% SynCAM4 (Fig. 6C,F,I).

### Expression of pre-synaptic organizers neuroligin-1 (NL1) and neuroligin-4 (NL4) in the adult spinal cord

**Figure 7:**
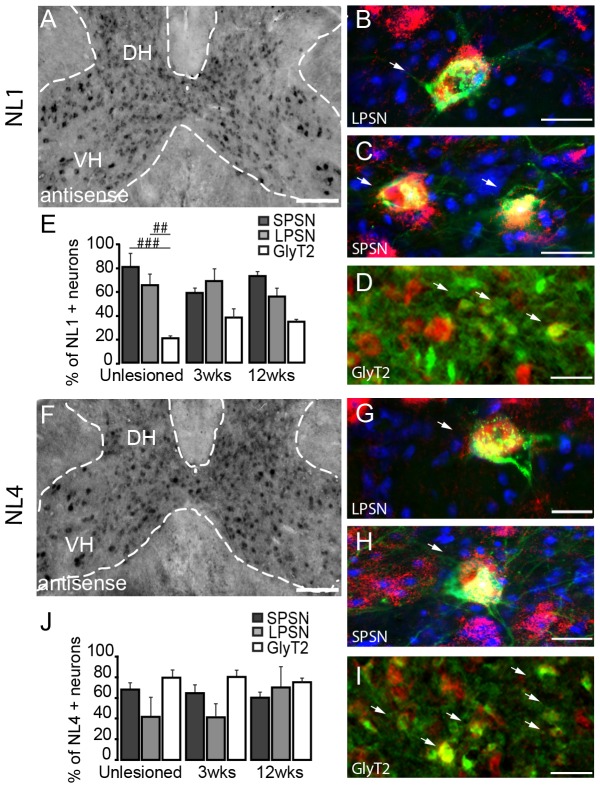
*In Situ* hybridization pattern of NL-1 and NL-4 in the cervical spinal cord. *In situ* hybridization of NL1 (A) and NL4 (F) mRNA in the unlesioned cervical spinal cord. Intense signals for NL1 (interneurons, A) and NL4 (inter- and motoneurons, F) are detected with the anti-sense probe. (B-D) Co-localization of NL1 in LPSN (B; LPSN: green; NL1: red; NT435: blue), SPSN (C; SPSN: green; NL1: red; NT435: blue) glycinergic neurons (D; GlyT2, green; NL1, red) in the cervical spinal cord. (E) Quantification of the number of GlyT2, LPSN and SPSN expressing NL1 in unlesioned and lesioned cervical spinal cord. (G-I) Co-localization of NL4 in LPSN (G; LPSN: green; NL4: red; NT435: blue), SPSN (H; SPSN: green; NL4: red; NT435: blue) glycinergic neurons (I; GlyT2: green; NL4: red) in the cervical spinal cord. (J) Quantification of the number of GlyT2, LPSN and SPSN expressing NL4 in normal and lesioned cervical spinal cord. DH: dorsal horn; VH: ventral horn. Arrows in D and I show double-labeled glycinergic neurons.Scale bar in A and F: 250 µm; Scale bar in B,C,G,H: 25 µm; Scale bar in D,I: 40 µm.

Neuroligins are postsynaptic adhesion proteins, which have been shown to promote synapse maturation and synaptic function [30]. Their receptors, the neurexins, have been shown to be widely expressed not only in development but also in the adult cortex and in particular in layer V where pyramidal cells reside [31]. We analyzed NL1 and NL4 expression in the cervical spinal cord of adult mice by *in situ* hybridization. Both NLs are strongly expressed throughout all laminae of the spinal gray matter (Table 2 and Fig. 7A,F). The analysis of different groups of spinal interneurons reveals that significantly higher percentage of long and short propriospinal neurons express NL1 compared to glycinergic neurons (Fig. 7B-E). In contrast no prominent differences in NL4 expression were observed between the different interneuronal populations studied (Fig. 7G-J). Further, the presence of a thoracic spinal cord injury did not change the expression pattern of either NL1 or NL4 in the cervical spinal cord (Fig. 7 I,J).

### Expression of bidirectional guidance and synaptogenic cues ephrinB1 and ephB2 in the adult CNS

**Figure 8:**
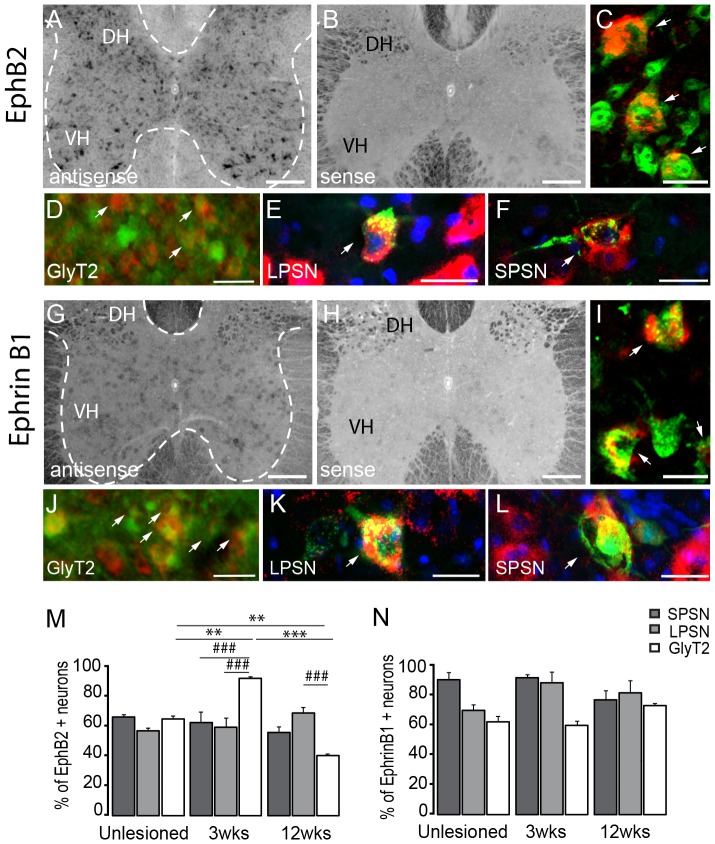
*In Situ* hybridization pattern of EphB2 and EphrinB1 in the cervical spinal cord. *In situ* hybridization of EphB2 (A), mRNA in the unlesioned cervical spinal cord. Intense signals for EphB2 is detected in inter- and motoneurons with the anti-sense (A). No signal is detected with the sense probe (B). (C) Confocal picture of double-labeled neurons in the ventral horn (NeuN, green; EphB2, red). (D-F) Co-localization of EphB2 in glycinergic neurons (D; GlyT2, green; EphB2, red), in LPSN (E; LPSN: green; EphB2: red; NT435: blue) and SPSN (F; SPSN, green; EphB2, red; NT435: blue) in the cervical spinal cord. *In situ* hybridization of EphrinB1 (G), mRNA in the unlesioned cervical spinal cord. Moderate signal for EphrinB1 is detected in inter- and motoneurons with the anti-sense probe (G). No signal is detected with the sense probe (H). (I) Confocal picture of double-labeled neurons in the ventral horn (NeuN: green; EphrinB1: red). (J-L) Co-localization of EphrinB1 in glycinergic neurons (J; GlyT2: green; EphrinB1: red), in LPSN (K; LPSN, green; EphrinB1, red; Neurotrace, blue) and SPSN (L; SPSN: green; EphrinB1: red; NT435: blue) in the cervical spinal cord. DH: dorsal horn; VH: ventral horn. Arrows in D and J show double-labeled glycinergic neurons.Scale bars in A, B,G,H: 250 µm; Scale bar in C,I: 25 µm; Scale bars in D,J: 40 µm; Scale bars in E,F,K,L: 25 µm.

**Figure 9:**
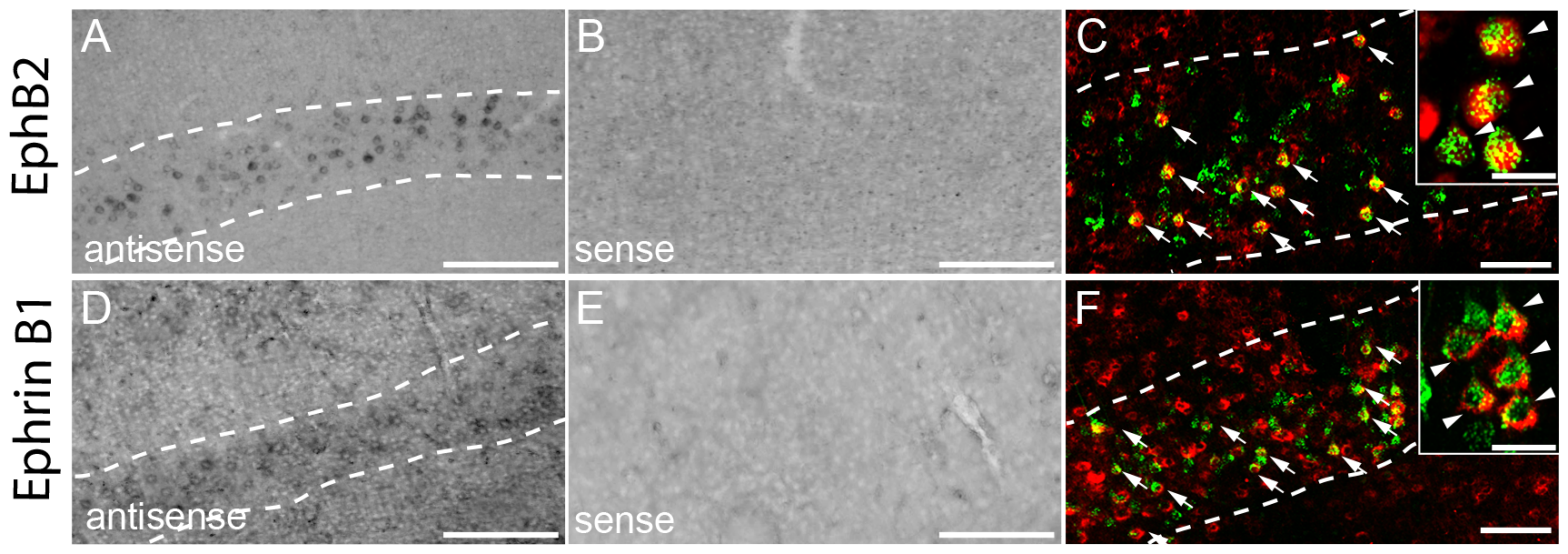
*In Situ* hybridization profile of EphB2 and EphrinB1 in cervical interneurons. *In situ* hybridization of EphB2 (A), EphrinB1 (B) mRNA in the cortex. No signal is detected with the sense probes for EphB2 (B) and EphrinB1 (E). Dotted lines in A and B represent layer V of the cortex. (C, F) Confocal images of double-labeled CST projection neurons (C; retrogradely-labeled CST projection neurons: green; EphB2: red and F; retrogradely-labeled CST projection neurons: green; EphrinB1: red). Scale bars in A, B, D, E: 100 µm; Scale bars C,F: 50 µm (25 µm in insets).

Ephrins and their receptors (Eph) are pleiotropic molecules involved in cell migration, axon guidance [32] and synapse formation [33] during nervous system development. Interestingly, eph-ephrin interactions can mediate both repulsive and attractive forces between cells [34]. EphB-ephrinB interaction has been shown to be important for proper ipsilateral targeting of CST and retinal axons [35]–[37]. *In situ* hybridization revealed that both ephB2 and ephrinB1mRNA are expressed throughout all laminae of the gray matter of the cervical spinal cord (Fig. 8A,B,G,H) with EphrinB1 having a dimmer expression in laminae I-IV (Table 2). Morphology analysis after counterstaining with NeuN suggests that both molecules are primarily expressed by neurons including by interneurons and at least in the case of EphB2 also motoneurons (Fig. 8C,I). Further characterization indeed showed that both EphB2 and Ephrin B1 are expressed in different interneuronal population located in the cervical spinal cord including LPSN, SPSN and glycinergic interneurons (Fig. 8D-F, J-L). While EphB2 mRNA is expressed by a similar proportion of these interneurons, Ephrin B1 was particularly prominently expressed in short propriospinal neurons in the healthy cervical spinal cord. Mostly, these expression patterns in the cervical spinal cord were not affected by the presence of a thoracic spinal cord injury (Fig. 8 M,N). The only exception is that the percentage of glycinergic interneurons expressing EphB2 was increased in the cervical spinal cord at 3 weeks post injury and decreased at 12 weeks after injury. Further expression analysis of cortical sections showed that both, Ephrin B1 and EphB2, were also expressed in layer II-III and layer V of the cortex (Fig. 9 A,B,D,E and Table 3). Retrograde labelling of layer V CST projection neurons revealed that 84, 6±3,5% of CST projection neurons express EphrinB1 and 74,1±2,2% EphB2 (Fig. 9 C,F).

## Discussion

While a number of the cues that determine the formation of neuronal circuits during the development of the nervous system have been identified, it is currently unclear which molecular signals can attract growing axon collaterals and initiate the formation of synapses during the remodelling of circuits in the injured adult CNS. The aim of this work was to study the expression of chemotropic and synaptogenic factors in the cervical spinal cord of adult mice to determine which of these cues are presented by spinal interneurons in the mature CNS. We focused our analysis on the cervical spinal cord – an area remote from the lesion site – as we and others have previously shown that new CST contacts with local cervical interneurons such as long propriospinal neurons play a key role during axonal remodelling following spinal cord injury [11]–[13]. While gene expression changes are certainly magnified at the lesion site [38]–[41], first reports indicate that a spinal lesion might also affect, although more moderately, gene expression changes more remote from the lesion site [38]; [41]; [42]. We mostly concentrated our efforts on membrane-bound molecules [7], [26], [43]–[47] as these are best suited to explain the attraction or repulsion of growing CST collaterals towards or from distinct spinal interneurons. To elucidate if and how the expression of these molecules in the cervical spinal cord changes following injury we analyzed the expression pattern at two time points following a midthoracic spinal cord injury. At 3 weeks after lesion when newly formed CST collaterals first initiate contact with spinal interneurons and 9 weeks later when these contacts have been refined [11], [13]. Our study now shows that (i) all the guidance and synaptogenic cues studied were not only expressed in the developing CNS but also expressed in the adult nervous system. (ii) While we did not detect cues that were exclusively expressed in a subpopulation of spinal interneurons, some molecules studied e.g. slits, Sema7a, SynCAM4 and NL1 were preferentially expressed in propriospinal neurons compared to glycinergic neurons. (iii) The expression pattern of guidance and synaptogenic molecules appeared to be stable over time and was by large not affected by a thoracic hemisection. It is interesting to note that the individual cues appear to be quite uniformly expressed throughout the different laminae of the adult spinal cord. This expression pattern is distinct from the region-specific pattern observed during neuronal development. For example, Semaphorin 3 mRNA is expressed between E13 and E 17 in the entire ventral half of the spinal cord but not in the floor plate [48]. Conversely, Slits are essentially expressed in the floor plate during development [49], [50]. Similarly it has been shown that expression of EphrinB ligands is confined to discrete regions of the spinal cord during development with for example EphrinB3 expression being localized to the floor plate around the ventral midline while EphrinB2 and B1 are primarily present in the dorsal spinal cord [51], [52]. The different regional expression pattern observed in the developing and adult spinal cord also indicates that the role of guidance and synaptogenic molecules might evolve in adulthood - classical repulsive cues in development might indeed become attractive in adulthood or conversely. While the exact role of these molecules in adulthood thus still needs to be better defined, their abundant expression suggests that they also can influence the formation and stabilization of circuits in the adult spinal cord. This view is supported by our finding that all receptors for guidance and synaptogenic molecules that we probed for were expressed in the cell bodies of CST projection neurons that reside in lamina V of the cortex. While we can formally only show that mRNAs are expressed in the neuronal cell body we believe that it is highly likely that functional receptors are present on growing CST axons as numerous studies in development show that the guidance and synaptogenic molecules that bind to these receptors factors can influence the behavior of CST axons [28], [35], [53]. In the following paragraphs, we summarize the expression pattern of the different families of chemotropic and synaptogenic cues and discuss their potential relevance in the context of the post-injury remodelling of axonal connections.

### Slit and Robo family

The attractive or repulsive action of axonal guidance cues in the developing nervous system has been documented extensively (for reviews see [54], [55]). One family of these guidance cues are the slit molecules (slit-1, slit-2 or slit-3) and their binding partners the Robo-receptors [56]. These molecules have, for example, been shown to prevent commissural neurons from re-crossing the midline in *Drosophila* [23]. More recently it has been suggested that slits are also expressed after spinal cord injury and can contribute to the failure of axon regeneration at the lesion site in the adult CNS [24]. We can show that slits are not only expressed at the lesion site [24], [56] but also in the cervical spinal cord of unlesioned animals. Their receptors in particular robo-1 and robo-2 are expressed throughout the forebrain while robo-3 is more sparsely expressed. This wide-spread expression of slits and robos in the adult CNS has previously been reported and suggests that these molecules also play an important role in the adult nervous system [57]. It is interesting to note that the slits were preferentially expressed by propriospinal neurons compared to glycinergic neurons. As propriospinal neurons are efficiently contacted by growing CST collaterals this might indicate that, in adulthood after injury, slit expression preferentially triggers neurite growth and arborisation rather than neurite repulsion as it has been shown for cortical interneurons during corticogenesis [58], [59].

### Semaphorin 6a and 7a and PlexinA2 and C1

Similarly to slits and robos, semaphorins and their receptors, the plexins [4], [28], [29], [60] have been implicated in the control of multiple aspects of neural development, including cell migration and axon guidance [61], [62]. In particular the transmembrane class 6 and 7 semaphorins, have been shown to be crucial regulators of axon growth, guidance and cell migration in many different parts of the brain [26]–[29], [62]. In this study we observed that Sema6a and Sema7a are expressed throughout the cervical gray matter. As previously reported, we found that plexinA2 and plexinC1, the respective receptors of Sema6a and Sema7a are expressed in the cortex in particular layer V where the cell bodies of the corticospinal tract resides [63], [64]. Interestingly, Sema7a is expressed by all propriospinal neurons but only some glycinergic neurons. As Sema7a is an attractive cue [63] that has been shown to promote axon growth [29] its expression might help to direct growing corticospinal collaterals towards propriospinal neurons during post-injury remodelling. A thoracic hemisection did not change the expression of semaphorin 6a and 7a in the cervical cord. This is in contrast to changes at the site of injury where Sema7a expression is increased in neurons, endothelial cells and components of the glial scar [65].

### SynCAMs

For circuits to remodel successfully growing collaterals not only need to reach their appropriate target cells but also form new synaptic connection. The formation of synapses requires the involvement of trans-synaptic adhesion molecules which span the synaptic cleft [66]. How the tight and precise alignment of the pre- and postsynaptic sites is achieved is currently under close investigation. In vertebrates this process is thought to be mediated via synaptic adhesion molecules. Synaptic Cell Adhesion Molecules (SynCAMs) comprise a group of four immunoglobulin (Ig) superfamily members that are crucial for the establishment of new synapses during development [66]. Interestingly these molecules are also prominently expressed in the adult brain [7], [66] and spinal cord [67], [68]. In line with these findings we detected the expression of SynCAM1, 3 and 4 throughout the cortex with a prominent expression in layer V of the cortex. SynCAMs are also present in interneurons and motoneurons in the spinal gray matter. In addition we detected SynCAM expression in the white matter which is consistent with expression in oligodendrocytes that has been reported by Thomas and colleagues [67]. While SynCAM1 and SynCAM3 were similarly expressed in all interneurons studied, SynCAM4 is preferentially expressed in propriospinal neurons. The latter is reminiscent of the differential presence of SynCAMs in excitatory and inhibitory neurons has been reported previously in the hippocampus [67]. Overall however the abundant expression of SynCAMs in the spinal cord suggests that while they might contribute to formation of synaptic contacts between CST collaterals and spinal interneurons [68] they are unlikely to explain the differential targeting and stabilization of contacts observed during intra-spinal remodelling.

### Neuroligins

Neuroligins [46], [47] and their presynaptic partners, the neurexins [69], [70], are another family of molecules that have been shown to regulate the maturation of functional synapses [30], [71], [72]. The expression of neurexins in the adult murine cortex has been previously documented [31]. In this study we now show that NL1 and NL4 are expressed throughout the cervical gray matter in both interneurons and in particular in the case of NL1 also in motoneurons. This is consistent with studies showing expression of NL2 and NL3 in spinal motoneurons [73]. Interestingly, NL1 was prominently expressed by propriospinal interneurons. As NL1 is known to be important for excitatory synapse formation [74] it might contribute to the establishment of mature synapses between CST collaterals and propriospinal neurons. Overall neuroligin expression in the neurons located distant from the lesion site in the cervical spinal cord did not changes substantially in response to a thoracic spinal cord injury while a downregulation of neuroligin mRNA expression has been previously reported to occur in transected neurons [73].

### Ephrin B1 and EphB2

Ephrins and their receptors (Eph) regulate synaptic function and eph-ephrin interactions can activate both repulsive and attractive forces between cells [32]. As a result these interactions can influence crucial aspects of nervous system development including cell migration, axon guidance, and topographic mapping [32]. Interestingly ephB-ephrinB interaction has been shown to control proper ipsilateral targeting in the visual system and in the developmental CST [35]–[37]. We now find that ephrinB1 and ephB2 are expressed in neurons throughout the spinal gray matter as well as in parts of the white matter. While expression of ephrinB1 and ephB2 in spinal interneurons has not been reported so far, expression in the white matter has already been shown to occur following SCI and expression of ephB2 has been reported in meningeal cells and of ephrinB1 and ephBs in astrocytes [75]. We also show that both ligands are expressed in layer II-III and V of the cortex consistent with previous reports of ephB2 expression in the brain [76], [77]. EphrinB1 and ephB2 were expressed in a large proportion of all interneurons studied both before and after spinal cord injury indicating that, similar to SynCAMs, ephrinB1-ephB2 interactions might contribute to the establishment of functional synapses but are unlikely to explain the differential stabilization of synaptic contacts during post-injury remodelling.

In summary, our systematic characterization of the expression pattern of guidance and synaptogenic molecules in the adult cervical spinal cord demonstrates that a large proportion of the cues that regulate developmental circuit formation are also present during the remodelling of circuits after injury. This suggests that many of the mechanistic insights gained by studying the developing nervous system might also help to better understand how the adult nervous system reacts to injury. Clearly, further studies are warranted to define the roles that each of these molecules play during the formation and maturation of new circuits after injury and ultimately to provide new avenues for the therapeutic support of axonal remodelling.
